# Bacterial etiology and antimicrobial resistance pattern of pediatric bloodstream infections: a 5-year experience in an Iranian referral hospital

**DOI:** 10.1186/s12879-024-09260-w

**Published:** 2024-04-03

**Authors:** Sadaf Sajedi Moghaddam, Setareh Mamishi, Babak Pourakbari, Shima Mahmoudi

**Affiliations:** 1 Pediatric Infectious Disease Research Center, Tehran University of Medical Sciences, Tehran, Iran; 2 Department of Infectious Diseases, Pediatrics Center of Excellence, Children’s Medical Center, Tehran University of Medical Sciences, Dr. Gharib Street, Keshavarz Boulevard, Tehran, Iran; 3 Biotechnology Centre, Silesian University of Technology, Gliwice, Poland; 4 Tehran University of Medical Sciences, Tehran, Iran

**Keywords:** Bloodstream infection, Antimicrobial susceptibility, Bacteria

## Abstract

**Background:**

Bloodstream infections (BSI) are the major cause of morbidity and mortality in children in developing countries. The purpose of the current study was to establish the antimicrobial susceptibility pattern of bacterial isolates from bloodstream infections at Children’s Medical Center Hospital (CMC), Tehran, Iran.

**Methods:**

We retrospectively recorded all positive blood cultures and antimicrobial susceptibility of all bloodstream isolates among children admitted to CMC, during 5 years. Specimen culture, bacterial identification, and antimicrobial susceptibility testing were performed according to standard laboratory methods.

**Results:**

From 3,179 pathogens isolated from the blood cultures 2,824 bacteria were cultured, with 1,312 cases being identified as Gram-positive bacteria (46%) and 1,512 cases as Gram-negative bacteria (54%). The most common Gram-negative bacteria isolated were as follows: *Pseudomonas* spp. (*n* = 266, 17.6%), *Klebsiella pneumoniae* (*n* = 242, 16%), *Stenotrophomonas maltophilia* (*n* = 204, 13.5%), Enterobacter *spp*. (*n* = 164, 10.8%), *Escherichia coli* (*n* = 159, 10.5%), *Pseudomonas aeruginosa* (*n* = 126, 8.3%), *Serratia marcescens* (*n* = 121, 8%), and *Acinetobacter baumannii* (*n* = 73, 4.8%). The most common Gram-positive bacteria isolated were coagulase-negative staphylococci (CONS) (*n* = 697, 53%), Streptococcus *spp*. (*n* = 237, 18%), *Staphylococcus aureus* (*n* = 202, 15%) and Enterococcus *spp*. (*n* = 167, 12.7%). 34% of bacterial strains were isolated from ICUs. The rates of methicillin resistance in *S. aureus* and CONS were 34% and 91%, respectively. *E. coli* isolates showed high resistance to cefotaxime (84%). All isolates of *K. pneumoniae* were susceptible to colistin and 56% were susceptible to imipenem. *P. aeruginosa* isolates showed high susceptibility to all antibiotics.

**Conclusions:**

Our findings emphasize the need of clinicians having access to up-to-date bacterial susceptibility data for routinely prescribed drugs. Continuous monitoring of changes in bacterial resistance will aid in the establishment of national priorities for local intervention initiatives in Iran. The increased risk of BSI caused by antibiotic-resistant organisms, emphasizes the significance of implementing appropriate antibiotic prescribing regulations and developing innovative vaccination techniques in Iran.

## Introduction

Bloodstream infections (BSI) pose a significant health challenge, particularly among children. These infections in the pediatric population are linked to undesirable levels of morbidity and mortality, highlighting the urgent necessity for robust diagnostic and therapeutic approaches [1]. Adding complexity to this challenge is the concerning trend of increasing antimicrobial resistance among the pathogens responsible for causing these BSIs [2–4].

Blood cultures play a pivotal role in the laboratory diagnosis of BSIs in infants and children, serving as the primary diagnostic method. The recovery of a pathogen from blood cultures holds significant advantages, as it not only confirms the diagnosis of bacteremia but also facilitates the identification of the specific pathogen and allows for susceptibility testing. This information is crucial for optimizing antimicrobial therapy in terms of both the choice of antibiotics and their duration [5].

To reduce antimicrobial resistance, healthcare professionals should consider proper antibiotic prescribing [3], as well as promote and enforce strong infection prevention and control measures in healthcare settings [6], such as hand hygiene, sterilization of medical equipment, and isolation precautions to prevent the spread of infections [7, 8]. Strengthening infection prevention and control measures in healthcare settings, coupled with promoting practices that minimize infection risks, will collectively contribute to reducing the spread of infections. Simultaneously, the implementation and enhancement of antibiotic stewardship programs in healthcare settings are vital to ensure judicious antibiotic use, with healthcare providers prescribing antibiotics only when necessary and based on accurate diagnosis [9]. The purpose of this study was to report the prevalence and antimicrobial susceptibility of blood culture isolates, track antimicrobial resistance rates and guide treatment decisions.

## Methods

This study was carried out retrospectively at the Children’s Medical Center Hospital (CMC), a tertiary referral hospital in Tehran, Iran, and one of the educational hospitals of Tehran University of Medical Sciences. In this study the isolates were collected during a five-year period from March 2015 to February 2019. During this five-year period, patients with positive blood cultures were detected and their information including age, gender, admission ward, culture result and antibiotic susceptibility of causative pathogens were collected [10–12].

The blood culture results and antibiotic susceptibility pattern were collected from the electronic database of the microbiology department of CMC. For children with multiple positive blood cultures during the same hospitalization, only one positive blood culture was included if the results were the same [13, 14].

The BACTEC 9120 Blood Culture System (BD, Franklin Lakes, NJ, USA) was employed for the rapid identification of microorganisms in blood samples. Subsequent to blood sample collection, Gram staining and subculture processes were conducted on MacConkey agar, chocolate agar, and blood agar plates. Complete microbiological identification relied on a combination of methods, including Gram stain, observation of morphological colonial characters (identifying hemolytic or non-hemolytic colonies on blood agar, lactose or non-lactose colonies on MacConkey’s agar), and the application of standard biochemical tests. Microorganism identification was accomplished through traditional biochemical methods, employing various tests such as Kligler iron agar slant, catalase, coagulase and oxidase tests, DNase, mannitol fermentation, sugar fermentation, Simmons’ citrate agar slant, urea hydrolysis slant, methyl red/Voges–Proskauer test, and motility tests [3, 15].

The antibiotic susceptibility of the isolates was assessed following the guidelines outlined by the Clinical and Laboratory Standards Institute (CLSI). Because of annual changes in the hospital formulary, antimicrobial agents utilized and tested varied from year to year. Data regarding the clinical significance of each isolate and whether the BSI was community or hospital-acquired were not available. The antimicrobial agents selected for analysis were those commonly used in the treatment of BSI in Iran [16–18]. In our study, intermediate susceptibility results were categorized as resistant to that antimicrobial agent.

### Statistical analysis

All the data obtained were entered in the Microsoft Excel worksheet and was analyzed using the Statistical Package for the Social Sciences (SPSS) software version 16 (SPSS Inc. Chicago, IL, USA). The data was characterized and summarized using descriptive statistics. GraphPad Prism 9.1.1 software was used to generate graphs.

## Results

During a five-year research period, the CMC recorded a total of 3,179 cases of positive blood cultures. Of these, 2,824 bacteria were cultured, with 1,312 cases being identified as Gram-positive bacteria (46%) and 1,512 cases as Gram-negative bacteria (54%). Male patients accounted for 57% (*n* = 1811) of the total cases (*p* value = 0.048). The age distribution of patients revealed a majority (*n* = 1842, 57.9%) under 1 year old, with 20.1% aged 1 to 5 years (*n* = 639), 12.4% between 5 and 10 years (*n* = 394), 8.2% in the 10 to 15 years category (*n* = 262), and 1.3% older than 15 years (*n* = 42).

**Fig. 1:**
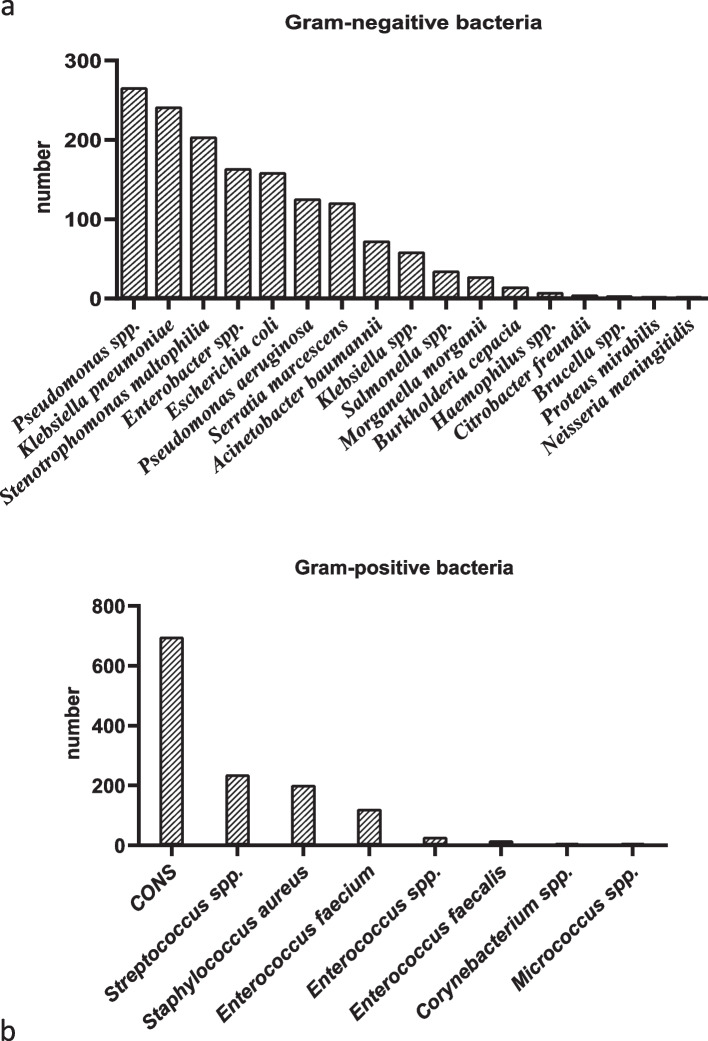
The distribution of Gram-negative and Gram-positive bacteria isolated from blood cultures of hospitalized patients throughout 2015–2019; **a** Gram-negative bacteria, **b** Gram-positive bacteria. CONS: coagulase-negative staphylococci

The occurrence of isolated bacteria fluctuated from year to year (*p* value ≤ 0.0001). According to the data provided, the highest percentage of isolated organisms belonged to the years 2016 and 2017, with each year accounting for 23% of the total isolated organisms. In 2018, the isolated organisms accounted for 18.2%, and in 2019, they constituted 18.6%. The lowest percentage of isolated organisms belonged to the year 2015, with only 17.3% of the total. The highest percentage of positive blood cultures was found in patients referred to the emergency room, accounting for 24%. Following that, the Intensive Care Units (ICUs) had the next highest percentage. The predominant Gram-negative bacteria isolated were as follows: Pseudomonas *spp*. (*n* = 266, 17.6%), *Klebsiella pneumoniae* (*n* = 242, 16%), *Stenotrophomonas maltophilia* (*n* = 204, 13.5%), Enterobacter *spp*. (*n* = 164, 10.8%), *Escherichia coli* (*n* = 159, 10.5%), *P. aeruginosa* (*n* = 126, 8.3%), *Serratia marcescens* (*n* = 121, 8%), and *Acinetobacter baumannii* (*n* = 73, 4.8%), respectively. The most common Gram-positive bacteria isolated were coagulase-negative staphylococci (CONS) (*n* = 697, 53%), Streptococcus *spp*. (*n* = 237, 18%), *Staphylococcus aureus* (*n* = 202, 15%) and Enterococcus *spp*. (*n* = 167, 12.7%), respectively (Fig. 1).

The distribution of isolated organisms across various years is presented in Table 1. The trends observed in the distribution of isolated organisms across the years 2015 to 2019 reveal dynamic patterns in the prevalence of specific bacterial species. The percentage of Pseudomonas *spp*. isolates increased consistently from 2.8% in 2015 to 22.3% in 2019, indicating a substantial rise in its prevalence over the years. The highest isolation rates for *S. maltophilia* and *S. marcescens* occurred in 2017 while the highest amount of isolation for *K. pneumonia* and *P. aeruginosa* strains was reported in 2018. CONS exhibited a consistent presence, comprising a significant portion of isolated organisms with a stable prevalence. *S. aureus* demonstrated relatively stable prevalence, with minor fluctuations from 13% in 2015 to 19% in 2019 (Table 2).

**Table 1:** The prevalence of Gram-negative bacteria isolated from blood cultures of hospitalized patients from 2015 to 2019

Organism	2015		2016		2017		2018		2019		Total	
	n	%	n	%	n	%	n	%	n	%	n	%
*Pseudomonas spp.*	13	2.8	64	9.8	66	17.8	49	19.9	74	22.3	266	17.6
*Klebsiella pneumoniae*	35	7.5	48	7.4	40	10.8	59	24	60	18.1	242	16
*Stenotrophomonas maltophilia*	19	4.1	90	13.8	88	23.8	0	0	7	2.1	204	13.5
*Enterobacter spp.*	20	4.3	27	4.1	59	15.9	18	7.3	40	12	164	10.8
*Escherichia coli*	37	7.9	39	6	23	6.2	23	9.3	37	11.1	159	10.5
*Pseudomonas aeruginosa*	23	4.9	16	2.5	16	4.3	38	15.4	33	9.9	126	8.3
*Serratia marcescens*	19	4.1	26	4	45	12.2	12	4.9	19	5.7	121	8
*Acinetobacter baumannii*	9	1.9	20	3.1	9	2.4	24	9.8	11	3.3	73	4.8
Klebsiella *spp.*	25	5.3	13	2	3	0.8	5	2	13	3.9	59	3.9
Salmonella *spp.*	8	1.7	3	0.5	10	2.7	8	3.3	6	1.8	35	2.3
*Morganella morganii*	1	0.2	1	0.2	0	0	0	0	26	7.8	28	1.9
*Burkholderia cepacia*	0	0	0	0	2	0.5	8	3.3	5	1.5	15	1
Haemophilus *spp.*	2	0.4	0	0	6	1.6	0	0	0	0	8	0.5
*Citrobacter freundii*	0	0	1	0.2	1	0.3	2	0.8	1	0.3	5	0.3
Brucella *spp.*	1	0.2	1	0.2	2	0.5	0	0	0	0	4	0.3
*Proteus mirabilis*	1	0.2	1	0.2	0	0	0	0	0	0	2	0.1
*Neisseria meningitidis*	0	0	1	0.2	0	0	0	0	0	0	1	0.1
Total	213		351		370		246		332		1512	

**Table 2:** The prevalence of Gram-positive bacteria isolated from blood cultures of hospitalized patients from 2015 to 2019

Organism	2015		2016		2017		2018		2019		Total	
	n	%	n	%	n	%	n	%	n	%	n	%
CONS	138	54	169	56	172	57	100	43	118	55	697	53.1
Streptococcus *spp.*	56	22	64	21	50	16	49	21	18	8	237	18.1
*Staphylococcus aureus*	34	13	37	12	43	14	47	20	41	19	202	15.4
*Enterococcus faecium*	0	0	24	8	37	12	34	14	27	13	122	9.3
Enterococcus *spp.*	28	11	0	0	0	0	0	0	0	0	28	2.1
*Enterococcus faecalis*	0	0	2	1	1	0	4	2	10	5	17	1.3
Corynebacterium *spp.*	0	0	5	2	0	0	0	0	0	0	5	0.4
Micrococcus *spp.*	0	0	1	0	1	0	1	0	1	0	4	0.3
	256		302		304		235		215		1312	

*CONS* coagulase-negative staphylococci

**Fig. 2:**
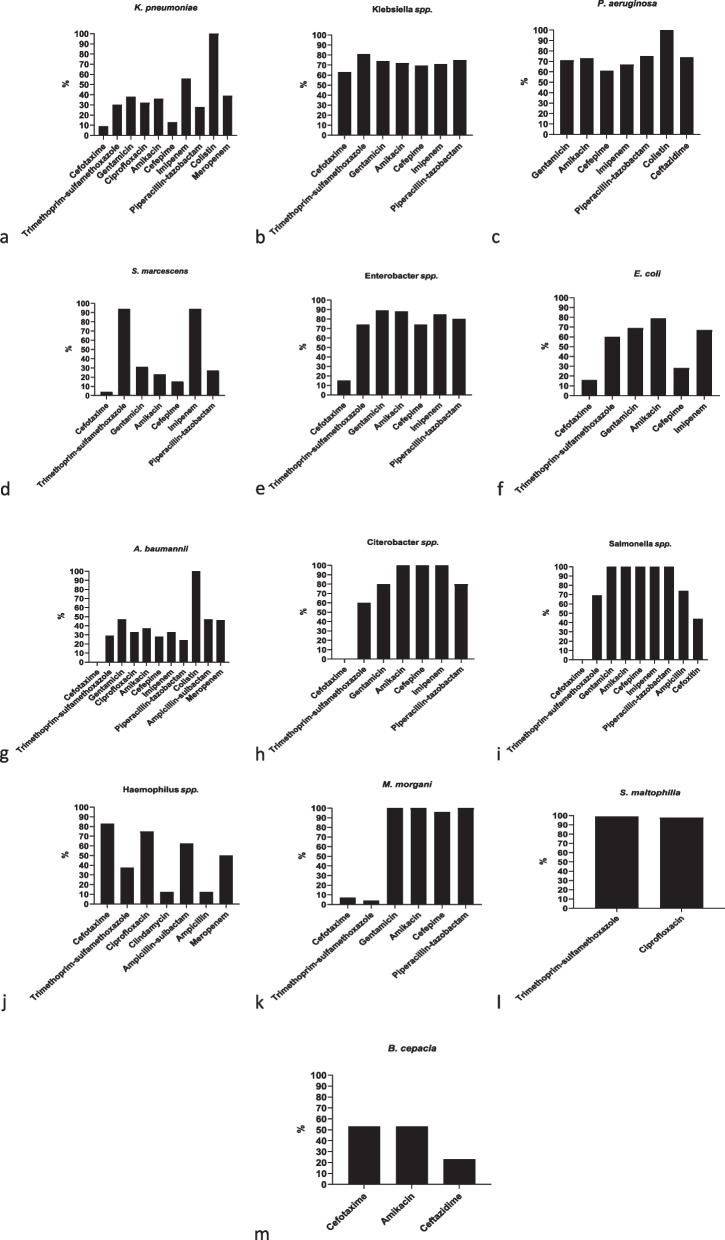
The sensitivity of Gram-negative bacterial species isolated from blood cultures to various antibiotics in Children’s Medical Center, Tehran, Iran. Each bar represents the number of bacterial isolates that are sensitive to the corresponding antibiotic

**Fig. 3:**
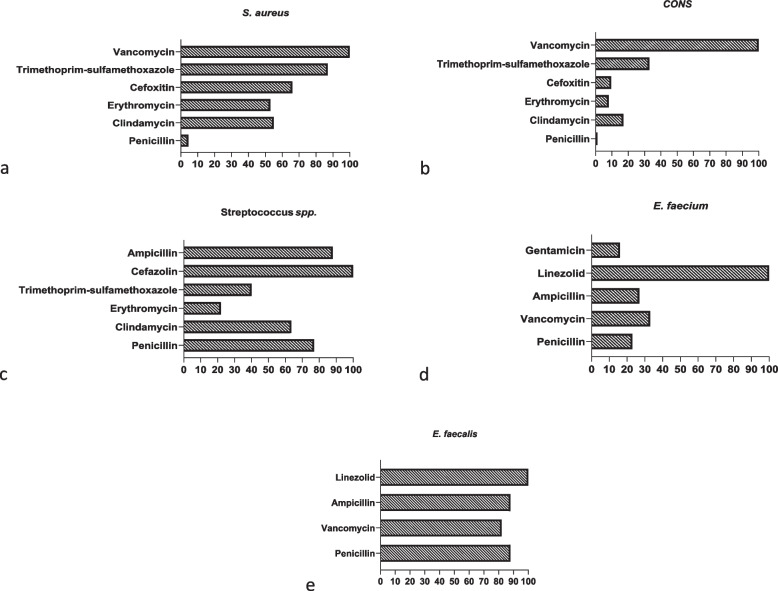
The sensitivity of Gram-positive bacterial species isolated from blood cultures to various antibiotics in Children’s Medical Center, Tehran, Iran. Each bar represents the number of bacterial isolates that are sensitive to the corresponding antibiotic. CONS: coagulase-negative staphylococci

Figures 2 and 3 shows the antimicrobial susceptibility rates for the Gram-negative and Gram-positive bacterial species recovered from blood cultures.

*K. pneumoniae* isolates showed high resistance to cefotaxime (91%), cefepime (87%) and piperacillin-tazobactam (72%); 56% were sensitive to imipenem and 100% to colistin. Klebsiella *spp*. isolates showed high sensitivity to trimethoprim-sulfamethoxazole (81%), piperacillin-tazobactam (75%), gentamicin (74%), and amikacin (72%). In *P. aeruginosa* isolates, the most sensitive antibiotics reported were colistin (100%), piperacillin-tazobactam (75%), ceftazidime (74%), amikacin (73%), and gentamicin (71%). *Morganella morgani* isolates showed 100% sensitivity to piperacillin, tazobactam, amikacin, and gentamicin. *Salmonella* spp. isolates had 100% sensitivity to piperacillin, tazobactam, imipenem, cefepime, amikacin and gentamicin. *S. marcescens* isolates showed high sensitivity to imipenem (94%) and trimethoprim-sulfamethaxazole (94%). *E. coli* isolates were 79% sensitive to amikacin, 69% to gentamicin and 67% to imipenem, while high resistance to cefotaxime was reported (84%). *A. baumannii* isolates showed high resistance to the piperacillin-tazobactam (76%), cefepime (72%), trimethoprim-sulfamethoxazole (71%).

Enterobacter *spp*. isolates were highly sensitive to gentamicin (89%), amikacin (88%), imipenem (85%) and piperacillin-tazobactam (80%). High resistance to cefotaxime was reported (85%). Citrobacter *spp*. isolates showed 100% sensitivity to imipenem, cefepime, and amikacin. Haemophilus *spp*. isolates demonstrated a high sensitivity of 83% to cefotaxime. However, these strains exhibited substantial resistance to clindamycin and ampicillin, with rates of 87.5% for both antibiotics.

Vancomycin demonstrated the highest efficacy against *S. aureus* isolates. Trimethoprim-sulfamethoxazole followed as the next most effective antibiotic, with a sensitivity rate of 87%. Notably, 34% of the isolated *S. aureus* were identified as MRSA (methicillin-resistant *S. aureus*). In the case of CONS, all isolates exhibited 100% sensitivity to vancomycin, while 91.5% showed resistance to methicillin and elevated resistance to other commonly used antibiotics. *E. faecalis* isolates displayed 82% resistance to vancomycin. Conversely, *E. faecium* isolates exhibited significant resistance to vancomycin (67%), while all strains remained sensitive to linezolid. For *Streptococcus* spp. isolates, high sensitivity rates were observed with cefazolin (100%), ceftriaxone (91%), and ampicillin (88%).

## Discussion

In our recent study of 2824 isolated bacteria, a distinctive shift in microbial composition was observed, with 46% categorized as Gram-positive and 54% as Gram-negative. This contrasts with a previous study conducted between 2010 and 2015 at the same center, where 59% of the 2325 isolated pathogenic bacteria from blood cultures were Gram-positive, and 41% were Gram-negative [19]. This comparison indicates a significant alteration in bacterial isolation patterns, signaling a discernible increase in the prevalence of Gram-negative bacteria in recent years. A thorough examination of historical data from earlier studies in the same center further underscores this evolving trend. In the periods 2001–2005 and 2006–2010, Gram-negative bacteria comprised 52.4% and 65% of isolates from blood cultures, respectively [10, 15]. Understanding this dynamic shift in bacterial composition is crucial for guiding healthcare strategies, particularly in terms of antibiotic prescription guidelines, the implementation of robust infection control measures, and overall optimization of bacterial infection management. Regular monitoring of these trends remains imperative for adapting healthcare interventions to effectively address the continually evolving microbial landscape.

The findings from our study highlight the prevalence of Gram-positive bacteria, with CONS being the most frequently isolated (53%), followed by Streptococcus *spp*. (18%), *S. aureus* (15%), and Enterococcus *spp*. (12.7%). In our study, the most common Gram-positive bacteria isolated were CONS (53%), Streptococcus *spp*. (18%), *S. aureus* (15%), and Enterococcus *spp*. (12.7%), while in the previous study done in CMC the most common Gram-positive organisms isolated were *S. epidermidis* (55.1%), *S. aureus* (19.5%), Enterococcus *spp*. (8.7%), *S. viridans* (6.5%), *S. hemolyticus* (5.1%), *S. pneumoniae* (2.9%), Group B Streptococcus (1.4%) and diphtheria (0.7%) [3]. In another study performed in CMC in 2001–2005, *S. aureus*, CONS, and *S. pneumoniae* were the most common Gram-positive organisms [15]. In the study done during the years 1995–2000 by Mamishi et al., the most common Gram-positive bacteria were CONS, *S. aureus*, Enterococcus *spp*., *S. pneumoniae*, and *S. viridans* [20].

Examining *S. aureus* across studies reveals its consistent prominence. In a Norwegian study spanning 2013–2017 [1], *S. aureus* emerged as the most common Gram-positive bacteria across all age groups. Similarly, a cross-sectional study in Nepal (2017–2018) reported *S. aureus* as the predominant Gram-positive organism, constituting 63% of isolates [21].

Studies across different regions consistently identify *S. aureus* and CONS as the predominant Gram-positive organisms in pediatric blood cultures [22, 23]. This consistency in the prevalence of specific Gram-positive organisms across diverse geographical locations emphasizes the global significance of these pathogens and underscores their clinical relevance in various healthcare settings.

In our study, the fact that 34% of bacterial strains were obtained from ICUs corresponds with the findings observed in the study conducted between 2010 and 2015 [19]. This consistency suggests a continued pattern of a substantial proportion of bacterial infections originating from ICUs over the years. Understanding and addressing the factors contributing to the prevalence of bacterial strains in ICUs are crucial for optimizing infection control measures and enhancing patient care in these critical environments.

It appears that in *S. aureus* strains, the highest reported antibiotic resistance was observed with penicillin at a rate of 95.5%. On the other hand, vancomycin showed 100% sensitivity, while trimethoprim/sulfamoxazole exhibited 87% sensitivity. The rates of resistance to clindamycin and erythromycin were reported as 45% and 47%, respectively, which aligns with the findings of a previous study conducted between 2010 and 2015 (3). Notably, 34% of the *S. aureus* in our current study were identified as MRSA, surpassing the previous study’s MRSA rate of 26% [3]. This heightened prevalence suggests a concerning trend in MRSA strains. In our study, CONS isolates demonstrated substantial resistance, notably with 100% sensitivity to vancomycin and a 91.5% resistance to methicillin. Comparatively, the 2010–2015 study highlighted similar resistance in *S. hemolyticus*, including complete resistance to penicillin and high rates for erythromycin (83.3%), and clindamycin (83.3%) [10]. Interestingly, no resistance to vancomycin was reported in either study, underlining its efficacy against CONS.

In this study,18% of *E. faecalis* strains were vancomycin resistant. A high sensitivity to penicillin and ampicillin was reported (88%). *E. faecium* strains showed high resistance to common antibiotics and vancomycin (67%); 100% of the strains were sensitive to linezolid. These susceptibility patterns were consistent with those reported in a previous study (3). The elevated resistance in *E. faecium* to various antibiotics, including vancomycin, emphasizes the complexity of managing Enterococcus infections.

In our study, the most common Gram-negative bacteria isolated were Pseudomonas *spp*., *K. pneumoniae*, and *S. maltophilia*. Previous studies from this center consistently reported *Pseudomonas* spp., Klebsiella *spp*. and *E. coli* as the most prevalent Gram-negative bacteria in blood cultures [10, 15, 20]. A notable aspect in our study was the elevated percentage of isolation of *S. maltophilia* strains, potentially attributed to nosocomial infections in the hospital, particularly in 2017. Cefepime was the most active of the cephalosporins against *E. coli* with 97.1% susceptibility rate and the carbapenems, imipenem and meropenem were 100% susceptible. On the other hand, susceptibility to aminoglycosides varied from 97.1% for amikacin to 88.0% for tobramycin [24]. In our study, *E. coli* strains were 79% sensitive to amikacin, 69% to gentamicin and 67% to imipenem. High resistance to cefotaxime and cefepime was reported (84%,72%, respectively). Contrasting with findings in Latin America where *P. aeruginosa*, the third most common Gram-negative pathogen, displayed extensive resistance to many antimicrobial agents, our study observed high susceptibility of *P. aeruginosa* to common antimicrobial agents [24]. *K. pneumoniae* showed elevated resistance to cefotaxime (91%), cefepime (87%), and piperacillin-tazobactam (72%), while 56% were sensitive to imipenem and 100% to colistin. While other Klebsiella isolates showed high sensitivity to trimethoprim-sulfomethoxazole (81%), piperacillin-tazobactam (75%), gentamicin (74%) and amikacin (72%). In *P. aeruginosa* strains, the most sensitive antibiotics reported were colistin (100%), piperacillin-tazobactam (75%), ceftazidime (74%), amikacin (73%) and gentamicin (71%). These findings align with the results obtained in the 2010–2015 and indicate lower resistance rates compared to reports from previous years [15, 20]. *S. marcescens* exhibited high sensitivity to imipenem and trimethoprim-sulfamethoxazole, aligning with previous studies [11, 25]. Haemophilus *spp*. isolates exhibited elevated sensitivity to cefotaxime, while demonstrating increased resistance levels to clindamycin and ampicillin compared to previous studies conducted in this hospital [11, 20]. *A. baumannii* isolates showed high resistance to all tested antibiotics, which was in consistent with previous studies conducted in this center [11, 19]. In the study conducted by Ballot et al., resistance to ceftazidime, ciprofloxacin, and gentamicin was 4%, 11%, and 15%), respectively, which was much lower than in our study [26]. In a study conducted by Qadeer et al. in Pakistan, the most prevalent Gram-negative pathogen was *A. baumanni* (15.3%) and this pathogen was 100% resistant to ceftazidim, ceftriaxone, ciprofloxacin, meropenem/Imipenem, and high resistance to other common antibiotics was seen [27]. These findings emphasize the persistent and concerning trend of multidrug resistance in *A. baumannii*, a pathogen known for its resistance to various classes of antibiotics, including beta-lactams and quinolones, with emerging resistance to aminoglycosides [28]. Enterobacter *spp*. isolates in our study exhibited high sensitivity to most common antibiotics, despite reporting elevated resistance to cefotaxime at 85%. This contrasts with the findings of a previous study in the same center, where 100% of Enterobacter *spp*. isolates were resistant to cefotaxime [11]. The observed change in resistance patterns highlights the dynamic nature of antibiotic susceptibility in bacteria over time and underscores the importance of ongoing surveillance and adaptation of treatment strategies based on current resistance profiles.

This study presents several limitations. The possibility of missing data is associated with its retrospective nature. Given that the data were gathered in a pediatric tertiary referral center, the proportion of children with complex illnesses is higher than that of the general population.

## Conclusion

Our findings emphasize the need of clinicians having access to up-to-date bacterial susceptibility data for routinely prescribed drugs. Continuous monitoring of changes in bacterial resistance will aid in the establishment of national priorities for local intervention initiatives in Iran. The increased risk of BSI caused by antibiotic-resistant organisms, emphasizes the significance of implementing appropriate antibiotic prescribing regulations and developing innovative vaccination techniques in Iran.

## Data Availability

The data presented in this study are available upon request from the corresponding author.
